# Visual analogue scales (VAS): Measuring instruments for the documentation of symptoms and therapy monitoring in cases of allergic rhinitis in everyday health care: Position Paper of the German Society of Allergology (AeDA) and the German Society of Allergy and Clinical Immunology (DGAKI), ENT Section, in collaboration with the working group on Clinical Immunology, Allergology and Environmental Medicine of the German Society of Otorhinolaryngology, Head and Neck Surgery (DGHNOKHC)

**DOI:** 10.1007/s40629-016-0006-7

**Published:** 2017-01-19

**Authors:** Ludger Klimek, Karl-Christian Bergmann, Tilo Biedermann, Jean Bousquet, Peter Hellings, Kirsten Jung, Hans Merk, Heidi Olze, Wolfgang Schlenter, Philippe Stock, Johannes Ring, Martin Wagenmann, Wolfgang Wehrmann, Ralph Mösges, Oliver Pfaar

**Affiliations:** 1 Center for Rhinology and Allergology, An den Quellen 10, 65183 Wiesbaden, Germany; 2 Charité Allergy Center, Department of Dermatology, Venereology, and Allergology, Charité, Berlin, Germany; 3 Department and Outpatient Clinic for Dermatology and Allergology am Biederstein, Technical University of Munich, Munich, Germany; 4 CHRU, Montpellier University Hospital Center, Montpellier, France; 5 MACVIA-LR, Montpellier, France; 6 INSERM, VIMA, Université Versailles St.-Quentin-en-Yvelines, Versailles, France; 7 Institute for Otorhinolaryngology, Leuven University Hospital, Leuven, Belgium; 8 Institute für Otorhinolaryngology, Academic Medical Center, Amsterdam, The Netherlands; 9 Group Practice for Dermatology, Erfurt, Germany; 10 Department of Dermatology and Allergology – Department of Dermatology, RWTH University Hospital, Aachen, Germany; 11 ENT Department, Charité University Hospital, Berlin, Germany; 12 Formerly: Otorhinolaryngology, St. Marien Hospital, Frankfurt, Germany; 13 Pediatric Pneumology and Allergology, AKK Altonaer Children’s Hospital, Hamburg, Germany; 14 Christine Kühne Center for Allergy Research and Education (CK-Care), Davos, Switzerland; 15 Ear, Nose, and Throat Department, Düsseldorf University Hospital, Düsseldorf, Germany; 16 Dermatology Group Practice, Münster, Germany; 17 Institute of Medical Statistics, Informatics and Epidemiology, Medical Faculty, University of Cologne, Cologne, Germany; 18 Department of Otorhinolaryngology, Head and Neck Surgery, Medical Faculty Mannheim, Universitätsmedizin Mannheim, Heidelberg University, Mannheim, Germany

**Keywords:** Allergic rhinitis, Treatment monitoring, Visual analogue scales

## Abstract

**Backround:**

Visual analogue scales (VAS) are psychometric measuring instruments designed to document the characteristics of disease-related symptom severity in individual patients and use this to achieve a rapid (statistically measurable and reproducible) classification of symptom severity and disease control. VAS can also be used in routine patient history taking and to monitor the course of a chronic disease such as allergic rhinitis (AR). More specifically, the VAS has been used to assess effectiveness of AR therapy in real life, both in intermittent and persistent disease.

**Methods:**

This position paper takes a detailed look at the historical development of VAS and its method-specific principles. Particular focus is put on aspects of practical application in daily routine and on a critical discussion of the advantages and disadvantages of the individual methods.

**Results:**

VAS are well validated for the measurement of AR symptoms and correlate well with the ARIA (allergic rhinitis and its impact on asthma) severity classification and also correlated well with rTNSS and RQLQ. Moreover, several treatment studies on AR have used VAS as an evaluation parameter. Thanks to the use of new (real-life and real-time) communication technologies, such as smartphone apps, Discussion: VAS can be used relatively simply and highly effectively to assess disease control. The VAS lends itself very well to digitization and has now been incorporated into a smartphone app (called Allergy Diary) to assess AR control and direct treatment decisions as part of an AR clinical decision support system (CDSS). MASK Rhinitis has developed this app, which is currently available in 15 different languages.

## Introduction

A detailed and thorough patient history combined with a physical examination of the patient while taking the reported symptoms into particular consideration form the focus of allergy diagnostics. When monitoring allergic rhinitis (AR), patient history in terms of the time, location, and situation in which symptoms occur plays an equally important role and should be supported by means of measuring parameters aimed at a semi-quantitative—or as quantitative as possible—rating of symptom severity and type.

The diagnosis of AR is established if two or more of the following symptoms have been identified using appropriate tests: nasal obstruction, secretion, sneezing, or nasal itching lasting for more than 1 h/day for more than 2 weeks/year, as well as an allergen-mediated cause of these symptoms [31, 32]. The type and severity of individual symptoms can vary from patient to patient and therapy should aim at total symptom control. Furthermore, it is essential in AR treatment monitoring to obtain reliable and comparable information relating to symptom severity before, during, and after treatment.

In order to document and evaluate these type of data, it is important that answers are not expressed in an arbitrary manner, but rather that they are assigned to a statistically documentable category. Such a categorization must enable both an intra- and interindividual comparison of AR symptom severity. To date, categorical scales have predominantly been used—primarily in controlled studies—to evaluate the efficacy of AR treatments, such as drug therapies and allergen-specific immunotherapy [1, 2].

However, these are poorly suited to daily routine, since they are prone to misinterpretation. Untrained test subjects do not use categories in four- to seven-level categorical scales as equally broad: the response options in the central categories are considered to be almost twice as broad (i. e., applicable) as the two outlying extremes. Thus, the linear assumption of equally broad categories only applies to the middle categories [3] and means that in a categorical scale users are much more likely to choose the middle categories. This bias can only be compensated by systematic training, which is unaffordable in routine patient care.

An AR VAS can represent a helpful alternative in this situation, since it is seen by test subjects as a continuum in which the same differences in symptom strength are assigned the same intervals on the VAS scale. Therefore, VAS is particularly suited for use in every day practice (by both patients and healthcare providers) since it is simple and intuitive to use (requiring no training), reproducible, sensitive, and suitable for everyday use.

Thus, particularly when combined with modern communication technologies such as the use of a smartphone app, a VAS can represent a valuable tool to document AR symptom severity and symptom control and can thus monitor treatment.

## Psychometric measurement instruments

In general, endpoints, at the very least, are assigned a verbal descriptor in psychometric scales to document subjectively perceived symptoms; this can potentially apply to each response category proposed in categorical scales. Scales need to be exhaustive in order to ensure that the entire spectrum of possible responses can be found between the endpoints.

In order to demonstrate that patients have the entire range of possible perceptions of symptoms at their disposal when responding, each end of the scale is defined with contrasting terms such as “always – never”, “applies completely – does not apply at all”, or “yes – no”. One end of the scale represents the maximum conceivable symptom strength (i. e., 100%), the other end no symptoms whatsoever (i. e., 0%).

## Visual analogue scales

Visual analogue scales (VAS) are psychometric response scales used to measure subjective characteristics or attitudes and have been used in the past for a multitude of disorders, as well as in market research and social science investigations, among others [3, 4]. VAS were first described in 1921 and referred to at the time as a “graphic rating method” [5]. The initial publication, which covered no more than one page, was presented as a new method for management personnel to evaluate the workers assigned to them [3].

Until the 1940s, only a handful of sociomedical and psychological publications addressed the topic of VAS. It was not until the 1960s that the literature showed rekindled interest in the use and study of VAS [3, 6].

The word “visual” in the term visual analogue scale emphasizes the concrete nature of this type of scale (straight line), in contrast to abstract, non-representable evaluation scales (“… I don’t feel well …”).

The word “analogue” stresses the infinitely variable, continuously changing response format. As a result, and particularly since the advent of computer technology, a verbal distinction to “digital” is made, whereby there is always a stepwise change per Bit.

VAS are, therefore, effectively classless, meaning that, theoretically, they permit an infinite number of gradations between endpoints—the variable is a latent continuum [7].

Thus, they clearly differ from (in the above sense, digital) categorical scales, which do not permit intermediate evaluations, and, in extreme cases, provide only two modalities (“yes” – “no”) [3].

VAS scores may also be classified retrospectively, by forming value groups. So for example in an Allergy Diary APP—those with VAS > 5 = uncontrolled AR, VAS 2–5 = partly controlled AR and VAS score < 2 = well-controlled AR. The process of linear category reduction, for example, may find application to this end [8].

One of the major advantages of VAS is that they are perceived as a continuum, meaning that their data are considered interval-scaled. Two equally sized intervals on a VAS are always interpreted as two equally sized differences by respondents. This makes it possible to calculate the arithmetic mean.

Data obtained from categorical scales, on the other hand, can only be interpreted in terms of their dissimilarity and rank; as such, the data are ordinal-scaled. Although the categories reflect a hierarchy, no statement can be made on how large the differences between the individual categories are for a respondent. Therefore, here it is only permissible to give median values.

## Practical application of visual analogue scales for AR

A VAS is usually a 100-mm long horizontal line with verbal descriptors (word anchors) at each end to express the extremes of the feeling. AR patients mark the point on the line that best corresponds to their symptom severity or AR control status. To this end, they are instructed to put a cross on the straight line at the point that most accurately expresses their degree of agreement.

When reading the VAS, the position of the respondent’s cross is generally assigned a score between 0 and 100. If documented in paper form, the scores can then be simply transferred to a 100-value scale using a millimeter tape measure. The division into hundredths is considered sufficiently sensitive [6].

When using electronic documentation options, analysis is usually performed in an automated manner by a programmed algorithm.

It is important to bear in mind when selecting verbal anchors that these are intended to verbalize the extremes in such a way that the entire spectrum of possible degrees is covered, and not only a part thereof [3, 4]. Thanks to the continuous response format, the patient is not restricted to a fixed number of potential responses, but instead the responses move along a continuum, a seamlessly coherent gradation.

The AR symptoms as a whole, as well as each symptom individually (e. g., nasal obstruction, rhinorrhea, itch, sneezing), can be evaluated on a separate VAS as well as the impact of AR on asthma for comorbid patients.

VAS should not have any markings (e. g., identifying the middle or dividing the line up into equally sized fragments), since the sensitivity of VAS without markings is higher than it is with [9]. The most important aspect of any VAS however is the question that is combined to it and not the line. The line remains the same but the question may change.

Numerous studies have shown that the respondent is guided by the principle of equisection, the cognitive subdivision into equal intervals [3, 4, 10]. As part of this, most users proceed in such a way that they first rate the scale endpoints that are clearly described by the verbal descriptors and which delimit the range described by the VAS. The middle of the scale, which is easily identified even without marking, is then rated. From the middle of the VAS line, it is possible to determine the respective midpoints to the two extremes, such that one can already identify five points (0, ¼, ½, ¾, 1). Working from these points, further differentiations are then made (by the user), so that the VAS can be sufficiently well divided without significant cognitive effort [10, 11]. Interestingly, methodological studies on the use of VAS showed a comparable approach among the majority of VAS users [3, 4, 10, 11].

Methods to construct equidistant, nonverbal category labels using VAS have been increasingly used recently, e. g., rating scales that use smilies as symbolic markings and verbal anchors [12].

VAS are used in most studies for individual comparisons, i. e., repeat measurements at different points in time, and as part of treatment monitoring [4].

## Advantages of visual analogue scales

The first detailed list of the advantages and disadvantages of VAS was published only 2 years after the concept was first described [13]. Although the findings in that article have been deepened and expanded upon, they still apply today given the general nature of the findings.

Perhaps the most obvious advantage of VAS is that they offer an extremely high degree of resolution and hence the option of very fine nuances of judgment [4, 5, 14]. The respondent is not bound in a potentially overly tight corset of predefined categories and, as a result, is able to express themselves more freely. That is arguably also the reason why individuals whose attitude lies between two categories prefer this type of scale [6, 8, 15]. Repeat measurements may indicate even minute changes [16, 17], which are nevertheless perceived by patients as already highly relevant in some cases [4]. The high degree of detail in VAS is, above all, an advantage in the case of items with low variance and permits rank-based tests to be used effectively. In the case of low-variance items, many cases fall into one category—hereby making them indistinguishable from one another—and are given the same position in the ranking. With VAS, even those cases that are extremely similar can be distinguished from one another [8]. The variation in significance of identical intervals on the VAS as interpreted by different users is thus smaller compared with categorical scales in which the values of individual categories fluctuate more among different users [8].

Moreover, from a subjective perspective, those tasked with processing the scales like using VAS, the main reason for this being their easy handling and decision-making [18]. Thus, it would appear that VAS are particularly well suited to routine treatment and have a positive effect on data quality. According to one study, VAS can have a moderating effect on socially desirable response behavior, since it is more difficult to estimate which value is expected on the scale continuum [8]. Thus, the values on VAS should be closer to the true attitudes of respondents compared with values obtained with categorical scales. Although, as with other types of scales, halo effects have been observed with VAS—a trend towards the middle was seen in particular when several items were to be evaluated—these were distinctly smaller than in categorical scales [16].

VAS permit statistically significant differences in distributions to be readily determined [6]. This type of scale is considered to be more accurate and sensitive and subject to less distortion and bias compared with categorical scales [19].

VAS are particularly well suited to measuring continuous features [20], as AR symptoms inherently are, since their continuous nature corresponds more to the score to be measured than is the case in graded categorical scales. This advantage with VAS proves to be true with other, entirely different subjective phenomenon, such as measuring mood, pain, and emotions [21]. A large number of studies have confirmed the reliability and validity of VAS measurements [6, 16, 22–27]. Data obtained with VAS can be converted parametrically to an interval-scale level [20].

Another advantage of VAS becomes apparent when one uses statistical test methods based on ranks for analysis. The ranks of items can be readily determined and the high data resolution results in a large number of possible ranks. The question of whether an odd or an even number of categories should be specified when constructing a response scale does not arise with VAS [3]. VAS data not only permit a more differentiated analysis of the middle categories, they also indicate slight tendencies in one direction. The problem whereby even respondents with average attitudes are forced to move towards an extreme no longer exists [3, 28].

## Disadvantages of visual analogue scales

The main practical disadvantage of VAS is that they require subsequent distance measurements [29]. This involves considerable effort in terms of data entry for statistical analysis, as well as high costs. However, electronic data entry (e. g., using a smartphone) gets around this problem. Furthermore, VAS can only be used in written (or digital) format and not for oral interviews. A minimum patient ability in terms of visual ability and hand-eye coordination is required in VAS [4].

One drawback encountered with VAS is that patients have difficulty finding the point on the line that best applies to them, i. e., weighing up the significance a distance from the verbal anchor has [14, 19]. This is the downside of dispensing with limiting categories. Although using VAS enables patients to make finely graded assessments, this can have negative effects if questions are unclear or patients feel ambivalent, given that clues on how to formulate an assessment are lacking.

Similar difficulties may be encountered when interpreting VAS scores. While it is easier to meaningfully interpret a modest number of categories, and verbal descriptors can be assigned to the respective categories, the interpretation of (raw) VAS scores is less clear. However, the question of the score from which a change on the VAS scale is to be considered clinically relevant and not seen simply as a random variation has now been well answered (see below).

## Visual analogue scales as a measurement instrument in allergic rhinitis

Objective methods to measure nasal obstruction include inspiratory peak flowmetry, acoustic rhinometry, and anterior rhinomanometry [30]. AR-specific as well as generic quality of life (QoL) questionnaires, with some including multiple items [33, 34], have been developed to assess QoL [31, 32].

These and other measuring instruments are undoubtedly suited to posing questions about the relevant parameters. However, around 80–90% of AR patients are cared for by their general practitioner and/or pharmacist or practice self-medication.

Thus, it would appear important to use a simple parameter that reacts readily to changes and that can be applied in different situations and at various levels of care. VAS are well suited for AR patient self-assessment both in specialist and general medical treatment [35, 36], as well as for treatment assessment by pharmacists [37]. Although VAS scores in AR do not differ significantly in terms of sensitivity and reproducibility from other psychometric tests using categorical scales [33, 38–43], they have proved to be superior in a number of studies in terms of user friendliness and better resolution of scores [21, 44].

Precisely because even very small changes are apparent with VAS, and these are sometimes more challenging to interpret compared with jumps on categorical scales, it is important to define the magnitude from which changes are considered relevant. There are extensive data on this for AR.

In most studies, a VAS of 50 (on a 100-mm scale) indicates moderate to severe AR [45–47]. Another study set the threshold at 60 mm [48]. Furthermore, VAS correlate well with the ARIA classification [39, 42, 43]. It was also noted that VAS scores that improved to below 50 mm as a result of treatment correlated well with a normalized rhinoconjunctivitis QoL questionnaire (RQLQ) and work productivity and activity impairment questionnaire: allergy specific (WPAI-AS) [38], whereas patients whose VAS remained above 50 mm continued to exhibit pathological scores in terms of QoL and work productivity [38].

It was shown for AR that, irrespective of the baseline VAS score, an improvement of 23 mm under therapy indicated effective treatment [49]; moreover, an improvement of 30 mm was always associated with improvements in QoL parameters [35, 49]. MACVIA ARIA defined AR control cut-offs are [35, 49]:

- >50: uncontrolled,
- 20–50: partly controlled,
- <20: well controlled.

Patients on placebo treatment, on the other hand, showed improvements of only 10 mm VAS in some studies [50, 51].

VAS were also used as an evaluation parameter in double-blind, placebo-controlled studies [50–56]. In two large AR studies to evaluate treatment outcomes with antihistamines, VAS were better able to discriminate between placebo- and verum-treated patients than could the total symptom score [50, 51].

VAS have also been successfully used in real-life and observational studies [52, 57, 58].

It was shown that VAS for AR can be used in all age groups—including preschool children (with supervision) [59], as well as elderly patients [60].

VAS have also been evaluated in numerous languages (e. g., German, French, English, Spanish, and Japanese) [43, 48, 50, 51, 60–63].

## Electronic collection of VAS data in AR

When a paper-based format is used, VAS readings (measuring the score with a ruler) takes up the greatest amount of time in terms of data analysis [3]. This time expenditure is dispensed with by means of automatically programmed analysis of online data, e. g., in the context of an app.

**Fig. 1:**
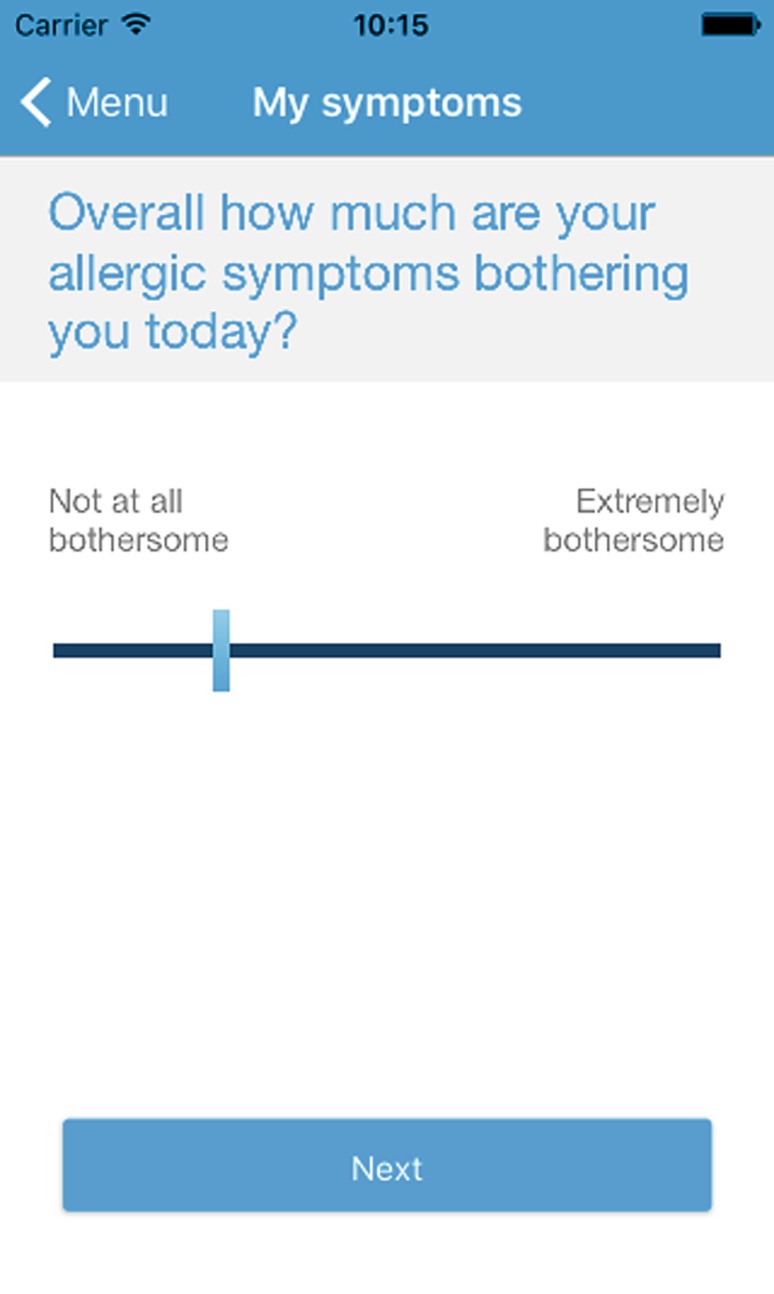
Practical application of visual analogue scales (*VAS*) in the MACVIA-ARIA app. (Users have to answer to the question indicated. When the user touches the indicated line, a marker bar appears. The marker bar can be moved backwards and forwards with a finger and placed at the appropriate point. Once the appropriate point on the line has been found, the user presses “Next” in order to proceed to the next VAS. Each VAS needs to be completed once daily)

The project group “MASK-Rhinitis” (MACVIA-ARIA Sentinel network) has developed a free smartphone app for patients called *Allergy Diary* and is currently developing a companion tablet-based app for healthcare providers (*Allergy Diary Companion*), both of which use a digitized VAS (Fig. 1; [58–60]). Daily symptom assessments are recorded in the MACVIA-ARIA app using a simple VAS, and VAS scores categorized as the degree of AR control. As recommended the VAS used is a simple line, with no incremental lines, with simple word anchors at either end. The question is “overall how much are your allergic symptoms bothering you today” from “not at all bothersome” to “extremely bothersome”.

The assessment scale is a VAS with the verbal anchors “not impairing” and “highly impairing” (separately for nose, eye, and asthma symptoms), which only need to be tapped in order to answer the questions. The app users are also asked to enter their daily medications they use. A reminder function helps patients to record symptoms regularly and to take their recommended treatment. Symptom assessments and drug requirements can be updated over the course of the day. Results (including those for longer time periods) are shown in graph form.

The aim of this app is to achieve rapid and sustained AR control and to make this measurable. This is intended to improve physician–patient communication. If a user records VAS scores indicative of poor AR control for 3 or more consecutive days, the app suggests that they seek medical advice.

Jean Bousquet et al. have tested and evaluated use of the app in real life, and the data obtained using the app, in a feasibility study. To this end, the data from the first 730 allergy diary users were tested.

Analysis of the results attested to the app’s high user friendliness, since all participants had answered the basic questions correctly. Furthermore, it was possible to identify simple phenotypical characteristics based on the app that helped rate rhinitis. For example, daily impairments, as well as impairments in working life, in patients with rhinorrhea appeared to be more pronounced the higher the number of symptoms noted by the user in the allergy diary app. However, the authors of the study concluded that their results need to be supported by the analysis of larger volumes of data obtained using the app (more app users), as well as further investigations. Also, it is not yet possible for the treating physician to evaluate the data, and possible patient concerns (e. g., data protection, central data collection, and commercialization) need to be discussed and resolved in the long term.

The *Allergy Diary* app is currently available in 15 different languages and can be downloaded for free in the Google Play Store and the Apple App Store. Similar electronic VAS diaries have already been used in numerous randomized, controlled studies [50, 51].

## Conclusion

A VAS represents a useful alternative to other psychometric scales for the documentation of AR symptoms and assessment of AR control.

A VAS is particularly suited to documenting AR symptom severity and disease control in routine treatment by virtue of its simplicity, time-saving handling and low susceptibility to errors. Since the VAS is seen by the patient as a continuum, the same differences in symptom severities are assigned the same intervals on the VAS.

Thus, particularly when combined with modern communication technologies such as a smartphone app, a VAS is a valuable tool for the documentation of symptom severity, treatment effectiveness, and disease control in AR.

## Abbreviations

AR: Allergic rhinitis
ARIA: Allergic rhinitis and its impact on asthma
MACVIA: MACVIA-ARIA sentinel network
QoL: Quality of life
RQLQ: Rhinoconjunctivitis quality of life questionnaire
VAS: Visual analogue scales
